# The metabolic plasticity of cancer stem cells: bidirectional crosstalk with organ-resident cells

**DOI:** 10.1038/s12276-026-01746-8

**Published:** 2026-06-09

**Authors:** Junseok Jang, Minseo Gwak, Hyunggee Kim

**Affiliations:** Department of Biotechnology, Korea University, Seoul, Republic of Korea

**Keywords:** Cancer stem cells, Cancer metabolism, Cancer microenvironment, Cancer therapeutic resistance, Drug development

## Abstract

Cancer stem cells (CSCs), defined as tumor cell populations with self-renewal and tumor-propagating capacity, contribute to tumor initiation and participate in progression, therapeutic resistance and relapse through pronounced metabolic plasticity. Although CSC metabolism has traditionally been regarded as a cell-intrinsic feature, accumulating evidence highlights the tumor microenvironment as a critical determinant of CSC metabolic states. Diverse stromal and tissue-specific parenchymal cell populations actively shape metabolic niches through context-dependent interactions, thereby reinforcing CSC stemness and adaptive potential. This Review synthesizes current insights into how widespread and organ-specific tumor microenvironment cell populations reprogram CSC metabolism via bidirectional crosstalk. Such a framework provides a mechanistic basis for intratumoral and organ-context-dependent heterogeneity, as well as differential therapeutic responses. Finally, we discuss the emerging potential of targeting CSC-supportive metabolic niches through drug repurposing, highlighting context-aware metabolic interventions as a pragmatic strategy to overcome CSC-driven treatment resistance.

## Tumor microenvironment shapes cancer stem cell metabolism

Cancer stem cells (CSCs) play a pivotal role in tumor initiation, progression, and resistance to therapy due to their remarkable metabolic plasticity. This review explores how CSCs adapt to diverse tumor microenvironments (TME) by reprogramming metabolic pathways, highlighting the potential of targeting CSC metabolism as a therapeutic strategy. The authors discuss the interactions between CSCs and tissue-resident cells alongside immune and endothelial populations in shaping the metabolic landscape of tumors. Key pathways, such as oxidative phosphorylation and fatty acid oxidation, that maintain CSC stemness and survival are discussed, including their bidirectional roles in mediating dynamic interactions between CSCs and the TME. The review also examines repurposing metabolic drugs for cancer therapy, given their established safety profiles and cost-effectiveness. It proposes integrating organ-specific metabolic traits with tumor and microenvironmental contexts to enable more precise metabolic interventions.

## Introduction

Tumors are heterogeneous diseases composed of hierarchical layers of cellular populations that function as a complex ecosystem. Driven by remarkable plasticity that enables genetic, phenotypic and functional reprogramming, cancer stem cells (CSCs) orchestrate tumor initiation, metastatic progression, therapeutic resistance and relapse, while adapting to and persisting in hostile conditions^1,2^. Despite their central role in tumor biology, establishing a precise, universally accepted definition of CSCs remains challenging, largely due to the lack of common markers consistently applicable across tumor types and organs. Therefore, in this Review, CSCs are operationally defined as tumor cell populations that possess self-renewal capacity while maintaining sufficient plasticity under microenvironmental or therapeutic pressures. Among the functional hallmarks that characterize CSCs, metabolic plasticity underpins the entire disease process, serving as a central force that enables CSCs to adapt and survive across diverse tumor microenvironments (TMEs).

**Fig. 1:**
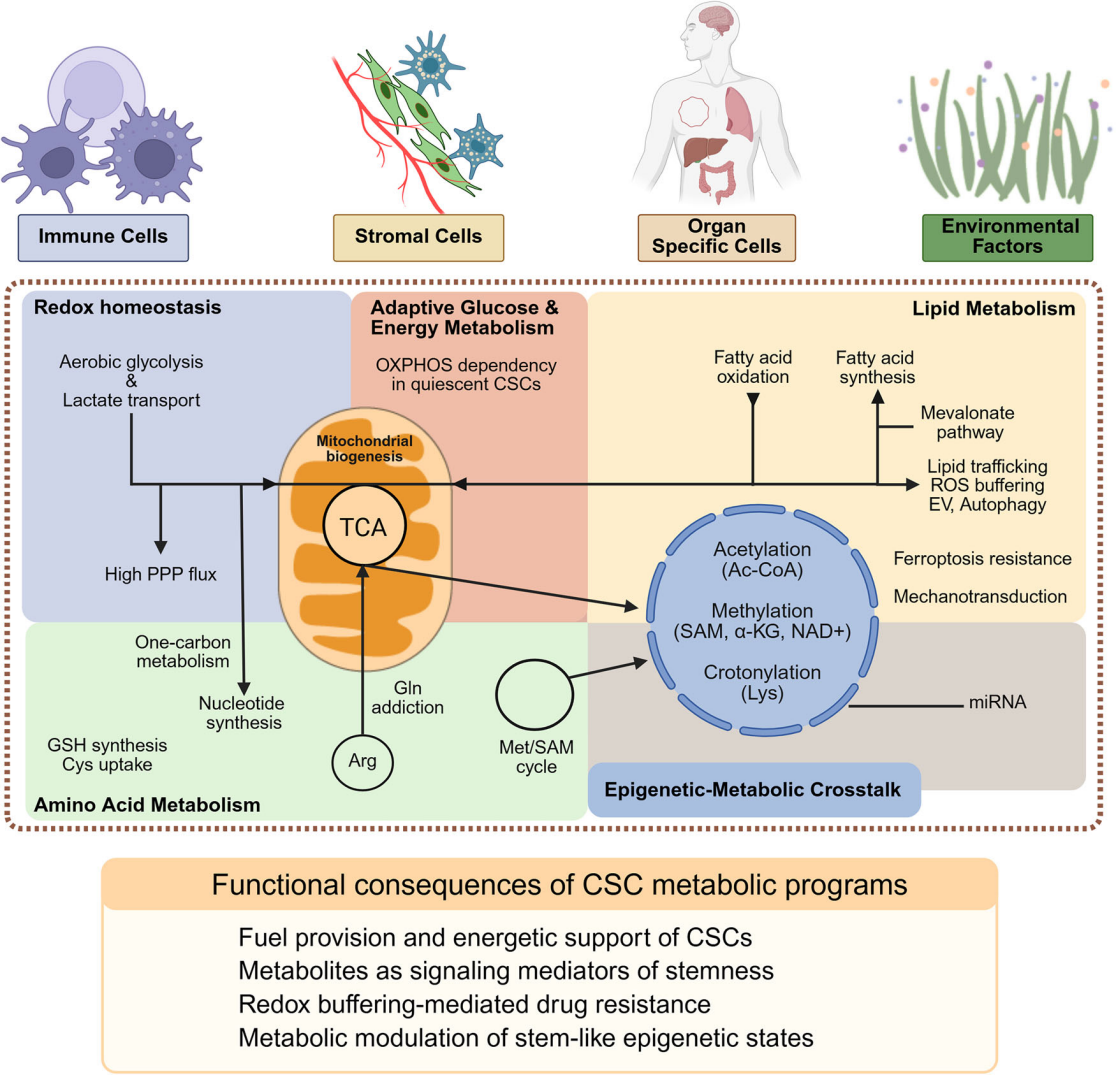
Metabolic plasticity supporting CSC adaptability and stemness within the TME. Within the TME, CSCs are exposed to various signals from immune cells, stromal cells, organ-specific parenchymal cells and other environmental factors. In response, CSCs actively engage in interconnected metabolic pathways, including redox balance, adaptive glucose and energy metabolism, lipid metabolism, amino acid metabolism and epigenetic–metabolic crosstalk, to support metabolic adaptation and maintain stemness. This figure offers a conceptual framework summarizing the key metabolic features of CSCs that collectively enable their adaptability and persistence within diverse TME. Ac-CoA, acetyl-coenzyme A; Arg, arginine; α-KG, α-ketoglutarate; Cys, cysteine; Gln, glutamine; Lys, lysine; Met, methionine; NAD, nicotinamide adenine dinucleotide; PPP, pentose phosphate pathway; SAM, *S*-adenosyl-L-methionine. Figure created in BioRender. Jang, J. (2026) https://BioRender.com/e9f60xu.

Targeting CSC metabolism therefore represents a promising therapeutic strategy, given its critical role in maintaining tumor-propagating potential and stem-like traits associated with malignancy (Fig. 1). In this context, repurposing metabolic drugs for cancer therapy offers a valuable opportunity for rapid clinical translation, supported by established safety profiles, cost-effectiveness, broad availability and suitability for long-term use. However, metabolic inhibitors exhibit variable anticancer efficacy across tumor types and often exert their effects through off-target mechanisms rather than their original modes of action. A recent review of clinical and preclinical repurposing studies reported that, among eight antihypertensive and six antidiabetic drugs evaluated for cancer therapy, only epalrestat demonstrated consistent on-target activity across multiple cancers by targeting AKR1B1/AKR1B10, the same molecular targets implicated in diabetes^3^. This limited efficacy of reliable metabolic targeting probably reflects both intratumoral heterogeneity and organ-context-dependent heterogeneity. In this Review, we address both intratumoral heterogeneity arising from CSC plasticity and niche interactions within individual tumors and organ-context-dependent heterogeneity, which reflects differences in metabolic programs across tumors originating in distinct organ environments. Consequently, the TME, particularly the metabolic interactions between CSCs and stromal as well as tissue-specific parenchymal cell populations, emerges as a key determinant of therapeutic response.

In this Review, we focus on how stromal and tissue-specific parenchymal cell populations actively reprogram CSC metabolism within the TME. By dissecting tissue-defined metabolic niches and their context-dependent effects on CSCs, we aim to provide mechanistic insights that may reveal actionable, tumor-type-specific metabolic vulnerabilities. Ultimately, elucidating the complex cell–cell interactions that regulate CSC metabolism is crucial for overcoming the inter- and intratumoral heterogeneity that currently limits effective treatment strategies.

## Ubiquitous TME cell types that drive intratumoral metabolic heterogeneity

**Fig. 2:**
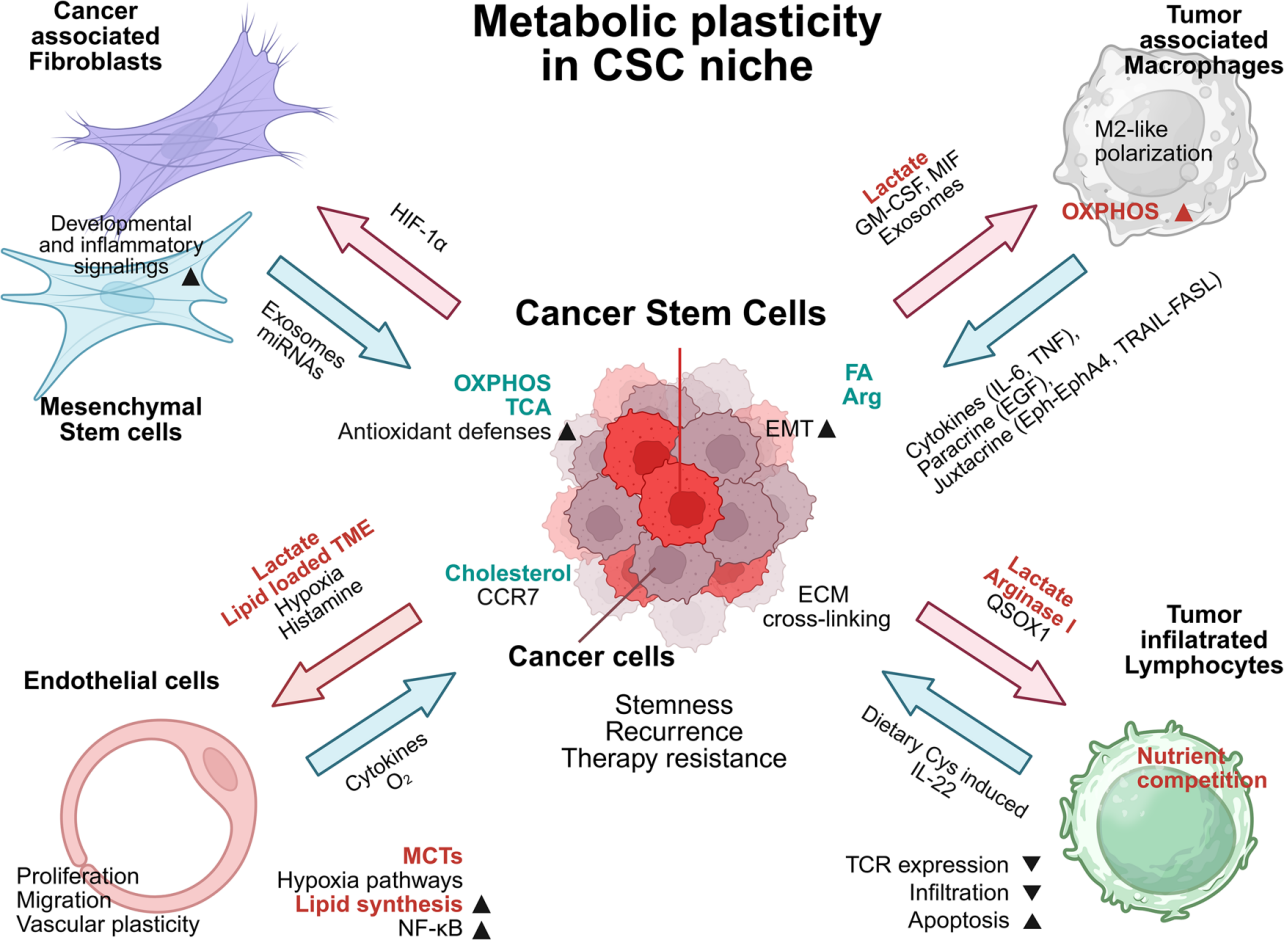
Metabolic plasticity within CSC niches influenced by common TME cell types. This schematic shows how CSCs establish and remodel the metabolic TME through dynamic interactions with common niche components, including TAMs, TILs, ECs, CAFs and MSCs. Red arrows and red-colored labels indicate the effects of CSCs on surrounding cell types, emphasizing how CSC-derived metabolites, cytokines and vesicular signals reprogram the immune, vascular and stromal compartments. Blue arrows and blue-colored labels show the reciprocal influences from niche cell groups toward CSCs, supporting stemness, EMT, recurrence and therapy resistance. Features related to metabolism are highlighted in bold, color-coded labels representing key metabolic pathways and dependencies, such as OXPHOS, TCA activity, lipid and amino acid metabolism, lactate shuttling, redox regulation and nutrient competition, which together define the CSC-supportive metabolic environment. CCR7, C–C chemokine receptor type 7; MIF, macrophage migration inhibitory factor. Figure created in BioRender. Jang, J. (2026) https://BioRender.com/3k17p5o.

Commonly observed across most solid tumors, specific TME cell types are broadly distributed throughout the body to systemically influence metabolic programs while simultaneously adapting to local tumor environments, thereby contributing to intratumoral heterogeneity. These cells perform essential functions—including immune regulation, stromal remodeling and angiogenesis—that collectively establish the metabolic framework of the TME (Fig. 2). Importantly, this framework is not static but is dynamically reshaped by spatial organization, metabolic stress and cellular interactions within tumors, resulting in localized metabolic niches that influence CSC states and therapeutic responses.

While bulk tumor studies typically address whether an individual metabolic pathway promotes tumor growth, research focused on CSC-centered metabolic interactions emphasizes the integration of metabolic and stemness programs. Accordingly, accumulating evidence highlights the importance of contextual diversity rather than universal principles, even among broadly shared TME cell types. In this section, we discuss recent studies through two complementary perspectives: CSC-driven reprogramming of TME cell metabolism and TME-mediated regulation of CSC metabolism and cellular state. Together, these reciprocal interactions reveal an interconnected CSC–TME metabolic architecture that deepens our understanding of how CSC metabolic niches are established, maintained and therapeutically exploited.

## Immune microenvironment

### Tumor-associated macrophages (TAMs)

Circulating monocytes differentiate into macrophages upon entering tissues and adopt diverse activation states, traditionally described as M1, which is pro-inflammatory and glycolysis-driven, or M2, which is associated with tissue repair and immunosuppression and relies on oxidative phosphorylation (OXPHOS). Within tumors, these macrophages are further reprogrammed into TAMs, which predominantly exhibit M2-like metabolic and functional characteristics that support tumor growth. Multiple studies demonstrate that, in many tumor contexts, CSCs in hypoxic, invasive or immune-modulating states favor glycolytic metabolism and lactate production^4–6^. The resulting lactate-rich TME promotes oxidative metabolism in macrophages via the pyruvate–OXPHOS pathway, thereby enhancing M2-like TAM polarization and immune evasion. In addition, under acidic conditions combined with granulocyte–macrophage colony-stimulating factor (GM-CSF) and macrophage colony-stimulating factor (M-CSF), this metabolic reprogramming has been shown to reinforce an inflammatory yet protumoral TAM phenotype^7^. Macrophage migration inhibitory factor, secreted by breast CSCs, induces metabolic reprogramming, increasing glycolytic activity in both tumor and immune cells, and thereby facilitating immune evasion^8^. Beyond lactate-mediated effects, CSC-derived exosomes can directly modulate metabolic enzymes and signaling pathways within TAMs, promoting macrophage polarization^5^. Simultaneously, iron-dependent lipid-peroxidation-driven cell death, known as ferroptosis, has been linked to broad remodeling of the immune microenvironment, including TAMs, toward a tumor-promoting state^9^. Collectively, these studies underscore that CSCs function as active metabolic regulators within the bulk tumor, shaping macrophage phenotypes and niches through metabolically driven secretory programs. Conversely, TAMs are widely recognized as key components of the TME that influence CSC states through multiple signaling pathways. TAM-derived cytokines, including interleukin (IL)-6 and tumor necrosis factor, activate nuclear factor (NF)-κB and STAT3 signaling to promote CSC maintenance, epithelial–mesenchymal transition (EMT), metastatic potential and therapy resistance. In addition, paracrine circuits, such as the EGFR/STAT3/SOX2 axis, further support the expansion of the CSC population^10–12^. Furthermore, juxtacrine interactions mediated by Eph–EphA4 or TRAIL–FASL signaling, along with macrophage–tumor cell fusion events, have been shown to directly contribute to CSC niche formation^13,14^. Recent studies have increasingly highlighted the significance of metabolic reprogramming in TAM–CSC interactions. TAMs modify CSC metabolic dependencies by altering fatty acid (FA) and arginine metabolism, as well as oxygen and nutrient availability, thereby creating conditions that favor CSC survival and stemness. Notably, TAM-induced increases in FA utilization and activation of the arginine–polyamine metabolic axis have been linked to pathways that directly support tumor development and CSC persistence^15^. Overall, these observations suggest that CSC-derived metabolic cues frequently initiate TAM reprogramming, highlighting CSC metabolism as a potential upstream target for disrupting this immunosuppressive niche^12^.

### Tumor-infiltrating lymphocytes (TILs)

Within the TME, CSCs drive metabolic remodeling—characterized by nutrient competition, lactate accumulation and a shift toward mitochondrial metabolism—to evade immune surveillance and ensure survival^16^. Activation of glycosphingolipid synthesis, observed in KRAS-driven cancers, serves as a key metabolic mechanism enabling CSCs to escape immune detection^17^, while metabolic barriers generated by enzymes such as QSOX1 promote extracellular matrix (ECM) cross-linking and physically block CD8^+^ T cell infiltration into dormant CSC niche^18^. The secretion of arginase I by CSCs and nearby TAMs depletes local L-arginine levels, which are essential for T cell activity, while simultaneously enhancing proline biosynthesis to promote collagen deposition, thereby downregulating T cell receptor expression^19,20^. Conversely, selective loss of mitochondrial complex I (NDUFS4/6) in cancer cells increases tumor immunogenicity and enhances TIL activity, highlighting CSC metabolism as a potential therapeutic vulnerability^21^.

Meanwhile, the metabolic environment of the TME influences the immune landscape, which, in turn, affects CSC plasticity. Metabolic inputs, such as dietary cysteine, can stimulate CD8^+^ T cells to release IL-22, which enhances stem cell activity through paracrine signaling, an interaction primarily described in intestinal stem cell models^22^. Beyond TIL dysfunction, tumor-derived lactate and acidification induce natural killer cell apoptosis, while heightened susceptibility of immune cells to ferroptosis further suppresses innate immunity, ultimately supporting CSC persistence and immune evasion^23–25^.

Collectively, the mechanisms described above, including nutrient competition, lactate accumulation, arginine depletion and metabolic barrier formation, indicate that CSC-driven metabolic remodeling of the immune microenvironment represents a dominant axis of immune evasion.

## Endothelial cells (ECs)

Enhanced glycolytic metabolism and lactate accumulation represent defining features of the hypoxic TME, providing a fundamental metabolic basis that promotes endothelial activation and angiogenesis. Importantly, both CSCs and vascular ECs rely on glycolysis to sustain their functions, a relationship that is well established^26^. In this metabolic context, lactate accumulated within the TME enters ECs through monocarboxylate transporters (MCTs), activating NF-κB–IL-8 signaling and hypoxia pathways involving HIF-1α and HIF-2α, thereby stimulating EC proliferation, migration and vascular plasticity in aggressive tumors^27,28^. In addition to glycolysis, CSC-driven lipid metabolic reprogramming further influences EC behavior. In glioblastoma, CSC-specific histamine secretion and lipid-derived mediators reprogram endothelial metabolism, leading to abnormal yet highly adaptable vascular networks^29–31^. Moreover, the reactivation of lipid synthesis pathways following withdrawal of anti-angiogenic therapy has been linked to tumor recurrence, indicating that metabolically plastic cancer cell subpopulations, including CSCs, contribute to this process^32^.

Conversely, ECs are increasingly recognized as metabolically active niche components that regulate CSC maintenance, stemness and adaptability. Recent studies using mass spectrometry imaging and single-cell transcriptomic analyses demonstrate that intratumoral lipid profile heterogeneity is associated with distinct microvascular architectures^33^, while also indicating that heterogeneity in endothelial metabolic states correlates with organ-specific CSC phenotypes and metastatic tendencies^34^. In addition, lymphatic endothelial-like cells promote glioblastoma stem cell growth through cytokine-driven cholesterol metabolism^35^.

Collectively, these observations support a reciprocal metabolic interaction between CSCs and ECs, suggesting that therapeutic strategies targeting both vascular metabolism and CSC metabolic plasticity may be required^36^.

## Cancer-associated fibroblasts (CAFs) and mesenchymal stem cells (MSCs)

CSCs actively remodel the stromal compartment through metabolically driven signaling, reprogramming fibroblasts and MSCs into tumor-supportive partners. CAFs and MSCs are mutually reinforced through the activation of developmental and inflammatory pathways, such as Notch, Wingless-related integration site (Wnt), fibroblast growth factor, platelet-derived growth factor, IL-1, NF-κB and transforming growth factor (TGF)-β, induced by CSCs, which are closely linked to cellular metabolic stress responses^37,38^. Notably, lactate functions not merely as a metabolic byproduct but as an HIF-1α-dependent paracrine signal that activates CAFs, which subsequently metabolically recycle CSC-derived lactate to sustain a fibrotic and immunosuppressive TME^39–41^. Similarly, tumor-recruited MSCs undergo metabolic reprogramming to support CSC-associated tumor growth, invasiveness and therapy resistance. Specifically, therapy-educated MSCs selectively enrich tumor-initiating cells, indicating that metabolically adapted stroma promotes recurrence and therapy resistance^42^. In leukemia, bone marrow MSCs support CSC survival under chemotherapeutic stress by maintaining mitochondrial OXPHOS and tricarboxylic acid (TCA) cycle activity while enhancing glutathione (GSH)-dependent antioxidant defenses^43^. In addition, MSC-derived exosomes deliver specific microRNAs that enhance CSC tumorigenic potential and stemness^44^. Overall, CSCs serve as metabolic organizers that convert fibroblasts and MSCs into a self-reinforcing stromal niche, thereby sustaining stemness, therapy resistance and tumor recurrence. This CSC-driven metabolic signaling may therefore represent a potential upstream therapeutic target.

## Organ-specific TME cell types refine tissue-dependent metabolic heterogeneity

**Fig. 3:**
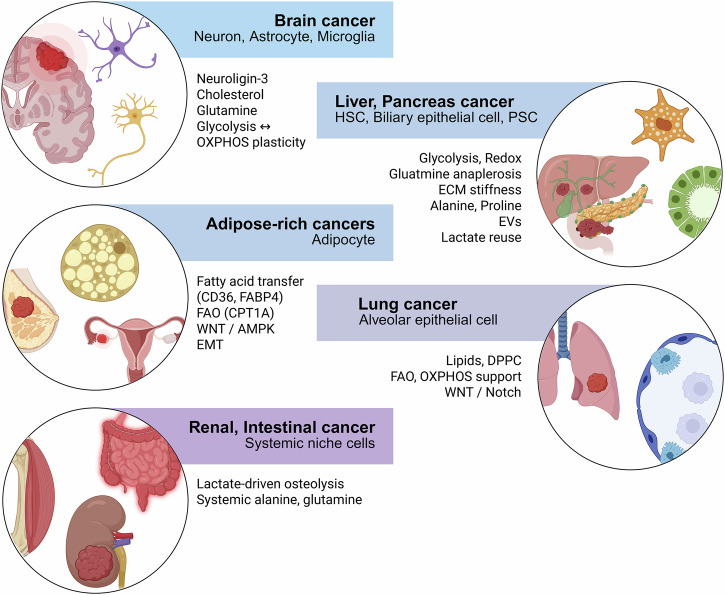
Organ-specific metabolic interactions between CSCs and tissue-resident niche cell types. This schematic summarizes the key metabolic interactions between CSCs and organ-specific parenchymal and stromal cell populations across different tumor types. For each organ system, tissue-resident niche cell types are grouped, and important metabolic features influencing CSC states are highlighted as keywords. Brain tumors focus on interactions among neurons, astrocytes and microglia, linking neuronal activity and glial metabolism to cholesterol, glutamine use and the flexibility of glycolysis–OXPHOS. Liver and pancreatic cancers depict fibrotic and stromal niches involving HSCs, biliary ECs and PSCs, characterized by redox regulation, amino acid anaplerosis, ECM stiffness and metabolite recycling. Adipose-rich cancers emphasize adipocyte-driven lipid transfer and FAO, while lung cancers highlight lipid-based support from alveolar ECs. Other contexts, including renal, intestinal cancers and systemic metabolic influences, are summarized together. DPPC, dipalmitoylphosphatidylcholine. Figure created in BioRender. Jang, J. (2026) https://BioRender.com/emtamh1.

Although common stromal cell types establish shared patterns of metabolic heterogeneity across tumors, accumulating evidence suggests that organ-specific microenvironmental cell types further refine the metabolic landscape in a tissue-dependent manner. These specialized stromal and parenchymal cells provide contextual metabolic signals that influence cancer cell adaptation, the CSC state and organ-level tumor behaviors (Fig. 3).

### Neurons

Recent studies demonstrate that tumor cells, including CSCs, actively remodel neural components within the TME and induce neurogenesis and axogenesis. Chemoattraction of nerve fibers and neurite outgrowth, mediated by tumor- and CSC-derived neurotrophic and axon-guidance signals, are common features of solid TME^45,46^. These processes overlap with developmental neurogenesis, as CSCs exploit conserved signaling pathways, such as NGF–Trk, Wnt and inflammation-related signaling^47,48^. Metabolic cues are also integrated into this process. For instance, dietary palmitic acid induces epigenetic reprogramming in cancer cells, leading to increased galanin secretion and subsequent activation of intratumoral Schwann cells, which generate a pro-regenerative ECM that supports metastasis^49,50^. Overall, CSCs actively coordinate tumor innervation by integrating developmental, inflammatory and metabolic signals.

Neural components within the TME act as key regulators of CSC maintenance, plasticity and malignant progression. Direct neuron–tumor interactions activate CSC-associated signaling pathways; notably, neuronal-activity-dependent secretion of neuroligin-3 stimulates PI3K–mTOR signaling, a central pathway linking growth control with anabolic and metabolic processes, thereby promoting glioblastoma proliferation and stemness^51^. Beyond soluble factors, tumor cells can electrically integrate into neural circuits through gap junctions and synapse-like structures, reinforcing undifferentiated, stem-like transcriptional programs^52,53^. Indirectly, neural signals modify the CSC-supportive niche by promoting angiogenesis and perineural invasion, both of which are associated with adverse clinical outcomes^54^. From a metabolic perspective, adrenergic nerves induce angiometabolic reprogramming that favors tumor growth, as observed in prostate cancer. These findings identify neural inputs as a regulatory axis linking CSC adaptation and tumor aggressiveness, underscoring the need for further investigation into the metabolic mechanisms underlying this axis.

### Glial cells—astrocytes and microglia

In glioblastomas, CSCs actively reprogram brain-resident glial cells, including astrocytes and microglia, shifting their normal functions toward a tumor-supportive state. Consistent evidence from in vivo infiltration analyses, spatial colocalization studies, in vitro co-culture systems and clinical prognostic data show the preferential accumulation of glial lineage cells near CSCs^55,56^. Within tumors, astrocytes and microglia enter reactive states that are closely linked to metabolic reprogramming. Reactive astrocytes change nutrient use and metabolic signaling to promote tumor invasion and immune suppression, whereas microglia undergo bioenergetic remodeling by shifting glycolytic and mitochondrial pathways to maintain activation and functional flexibility within the TME^56,57^. Reactive astrocytes supply metabolic substrates such as glutamine and cholesterol and control ABCA1-mediated cholesterol efflux, supporting tumor survival and reinforcing CSC-supportive metabolic niches^58^. Astrocyte-derived cholesterol signaling is also associated with the recruitment and polarization of TAMs^59^. Microglia show similar phenotypic plasticity, with M1-to-M2 transitions, whereas tumor-intrinsic changes, such as IDH1 mutations, alter microglial immune and metabolic programs, further biasing the microenvironment toward CSC support^60^. In addition, the regulation of ATP-binding cassette transporters, metabolic competition and exchange of metabolites between glial cells and CSCs contribute to therapy resistance and maintenance of stemness^61^. Collectively, these observations suggest that glial-derived metabolic support plays a dominant role in maintaining CSC-associated states in brain tumors, highlighting niche-directed therapeutic strategies.

### Hepatic parenchymal and stromal cells

In hepatocellular carcinoma, CSCs specifically remodel the parenchymal and stromal TME, characterized by communication through secreted factors and reprogramming of the fibrotic metabolic niche. Liver CSCs release extracellular vesicles (EVs) enriched with miRNAs such as miR-21, which can be taken up by hepatic stellate cells (HSCs) and promote their activation into a CAF-like phenotype^62^. Activated HSCs secrete pro-angiogenic and profibrotic cytokines, thereby strengthening the tumor-supportive environment. In addition, stem-like tumor cells influence biliary ECs by promoting inflammatory and progenitor-like states via JAG1-Notch, tumor necrosis factor and IL-6 signaling, thereby modulating the epithelial identity within the tumor^63^. Together, these CSC-driven programs promote ECM remodeling, development of the vascular environment, and metabolic changes in the surrounding hepatic cells, creating a supportive environment that facilitates tumor growth and adaptation.

From a reciprocal perspective, hepatocytes, biliary ECs and liver-resident stromal populations contribute to maintaining CSC survival and plasticity. Activated HSCs secrete periostin and deposit collagen- and laminin-rich ECM, generating stiff and hypoxic niches that sustain CSC quiescence, chemoresistance and tumor-initiating capacity^64–66^. HSC-derived EVs, including palmitoylated hexokinase 1, enhance glycolysis, proliferation and migration of CSCs, whereas in vivo cotransplantation studies have shown increased tumor growth and CSC marker expression^67^. Biliary ECs metabolically support CSCs via glutamine-dependent anaplerosis, which promotes mitochondrial metabolism and therapy resistance, especially in cholangiocarcinoma^68^. Additional stromal contributions, such as EVs from CAFs, adipocytes, TAMs and regulatory T cells, deliver FA, acylcarnitines, glycolytic enzymes and immunosuppressive signals that reinforce CSC stemness and immune evasion^69^. These multilayered metabolic pathways provide a basis for the growing interest in metabolic-targeting agents, such as metformin, as adjunct therapies for hepatocellular carcinoma and suggest that liver-specific CSC metabolic niches may influence therapeutic responses^64^.

From a therapeutic perspective, these observations indicate a metabolically reinforced reciprocal interaction between CSCs and hepatic niche cells, suggesting that effective therapeutic strategies may require targeting both tumor metabolism and the surrounding stromal environment.

### Pancreatic stromal cells

In pancreatic ductal adenocarcinoma (PDAC), CSCs depend on a stromal niche composed mainly of pancreatic stellate cells (PSCs) and CAFs to survive severe nutrient deprivation. PDAC cells that take up lactate through MCT1 support OXPHOS via mitochondrial metabolism and promote sphere formation and tumor growth^70^. CSCs induce autophagy in PSCs through signals, such as SHH, TGF-β and reactive oxygen species (ROS), directing PSC-derived alanine into the pyruvate–TCA cycle to reduce reliance on glucose and glutamine while supporting self-renewal^71^. In this context, PSCs and CAFs show increased glycolytic activity and secrete lactate, which CSCs reuse as oxidative fuel in a reverse Warburg-like metabolic system that maintains stemness^70^. ECM remodeling also enhances this niche by forming collagen-rich matrices that supply proline to fuel TCA flux, sustain redox balance and confer resistance to cell death. This metabolic crosstalk intersects with an acidic TME that promotes immunosuppression in PDAC, along with increased expression of immunosuppressive pathways, such as PD-L1 and the CD39–adenosine pathway, further supporting immune evasion^72^. Although the direct interactions between acinar cells and CSCs are not fully understood, hyperinsulinemia-driven activation of insulin receptor signaling in acinar cells causes metabolic overload, endoplasmic reticulum stress and the production of inflammatory cytokines, suggesting that reprogramming of acinar cells might create a microenvironment conducive to CSC emergence during PDAC initiation^73^. Overall, these metabolite-based symbiotic interactions and the immune landscape provide a core niche that enables tumor persistence and adaptation amid chronic nutrient scarcity and immune evasion within the PDAC TME. In this context, stromal metabolic support is a key determinant of CSC survival in PDAC, highlighting stromal-to-tumor metabolic coupling as a promising therapeutic target.

### Tumor-associated adipocytes (TAAs)

Adipocytes are stromal cells that substantially contribute to shaping CSC states by regulating lipid flux and FA oxidation (FAO), which are key metabolic pathways that support stemness in breast, ovarian and colorectal cancers^74–76^. Combined in vivo findings from tissue infiltration and spatial colocalization studies, along with in vitro co-culture systems and clinical correlations, link adipocyte-rich niches to increased tumor stemness, aggressiveness and poor prognosis. Notably, obesity and high-fat diet conditions further amplify these effects, as shown by expanded Lgr5^+^ intestinal stem cell populations and increased self-renewal through PPAR-δ-dependent metabolic signaling in colorectal cancer models^76^. From a CSC-centric perspective, CSCs actively remodel surrounding adipocytes into TAAs through inflammatory and stress-related cues, including IL-6, tumor necrosis factor and TGF-β, thereby inducing lipolysis, lipid droplet mobilization and FA export^77^. From adipocytes to CSCs, FAs released from TAA are taken up by CSCs via CD36 and FABP4 and then channeled into CPT1A-dependent FAO, supporting OXPHOS, redox balance and survival under glucose-scarce conditions^72,78^.

Meanwhile, adipocyte-derived adipokines, proteases such as matrix metalloproteinases, and metabolic intermediates activate key stemness-related pathways, including Wnt/β-catenin and AMP-activated protein kinase signaling, thereby supporting CSC self-renewal and EMT plasticity. In addition to FAs, adipocytes enhance glutamine utilization and GSH-dependent stress tolerance, further shielding CSCs from metabolic and therapeutic stresses^79^. Overall, adipocytes serve not only as passive lipid reservoirs but also as dynamic metabolic partners that provide fuel, redox capacity and signaling cues to sustain CSC metabolism and stemness. Collectively, these observations suggest that adipocyte-derived lipid metabolism strongly influences CSC metabolic adaptation, highlighting FA-related pathways as potential therapeutic vulnerabilities.

### Alveolar ECs

Lung metastasis has been thoroughly studied owing to its highly vascularized structure and immune microenvironment. However, the role of parenchymal cells, especially alveolar ECs, which are the most abundant cells in the lungs, has been relatively poorly examined. Among these cells, alveolar type 2 (AT2) cells perform specialized alveolar epithelial functions and exhibit stem cell-like properties with notable plasticity. Recent studies have shown the reciprocal activation of stem-like programs between metastatic cancer cells and AT2 cells within the lung metastatic niche^80,81^. In vivo models of breast cancer, melanoma and pancreatic cancer metastasis demonstrate the spatial colocalization of tumor cells with AT2 cells, along with increased stemness signatures in cancer cells and reprogramming of AT2 cell states^82^. Mechanistically, CSC-derived Wnt and TGF-β signaling have been shown to induce AT2 cell reprogramming, while AT2 cells, in turn, support CSC survival and growth through Wnt3a- or Notch-related signaling^80,83^. Indirectly, AT2-derived GM-CSF may contribute to an immunosuppressive microenvironment by promoting macrophage polarization^84,85^. From a metabolic perspective, AT2 cells secrete lung-specific lipids, such as dipalmitoylphosphatidylcholine and phosphatidylglycerol, which can be taken up by CSCs via lipid transporters, such as CD36, FABP4 and FATP2, potentially enhancing FAO and OXPHOS^86^. Overall, AT2 cells act as epithelial partners in the lung metastatic niche, providing structural and signaling support, as well as lipid-based metabolic inputs, thereby increasing CSC plasticity and promoting adaptive competence during lung colonization.

### Bone and muscle contexts

Beyond epithelial tissues, metabolite-driven niche interactions are also visible in mesenchymal and systemic contexts, especially in the bone and muscle environments. In the bone microenvironment, tumor-related acidification and lactate accumulation promote osteoclast activation through lactate uptake and oxidative metabolism, while simultaneously reducing osteoblast activity. This creates a permissive osteolytic niche that supports metastatic growth and stem-like programs in a cancer type-dependent manner^87–89^. Conversely, evidence for myocytes acting as direct CSC niche partners is limited; however, skeletal muscle plays a key role in systemic metabolite balance. During cancer-related metabolic stress, such as cachexia, myocyte-derived lactate, alanine and glutamine enter circulating nutrient pools, which may indirectly enhance tumor metabolic plasticity and stem-like fitness^90,91^. These findings support a broader perspective in which various parenchymal cell types, even those outside traditional stem cell niches, can influence CSC adaptation through localized or systemic metabolic inputs.

### Other epithelial and parenchymal metabolic niches

This section summarizes the additional epithelial and parenchymal cell types that support CSC metabolism across various tissues. In renal cell carcinoma (RCC), metabolic reprogramming within renal tubular epithelial compartments and tumor cells is marked by increased glycolysis and lactate accumulation, facilitating a lactate shuttle in which lactate is exported via MCT4 from glycolytic areas and taken up by MCT1-expressing tumor cells to fuel oxidative metabolism and promote stem-like fitness. In clear cell RCC, tumor-associated pericytes have been shown to secrete methionine, which promotes CSC survival and stemness, emphasizing a metabolite-driven perivascular niche^92^. In the intestinal epithelium, Paneth cells and intestinal ECs form a classic stem cell niche by providing Wnt3a and epidermal growth factor signals that are essential for maintaining intestinal stem cells and CSCs. Metabolic factors have also been reported to influence niche function^93,94^. Paneth cell-associated metabolic programs, including ROS management and diet-responsive ketone body signaling, support FAO and OXPHOS and strengthen stemness beyond traditional growth factor pathways^81^.

## Targeting the CSC-supportive metabolic niche through drug repurposing

Accumulating clinical and experimental evidence suggests that targeting CSC metabolism is a promising therapeutic approach, with drug repurposing of commonly used metabolic agents becoming particularly attractive^3,95,96^. This approach leverages well-established safety profiles and pharmacokinetics, thereby accelerating clinical translation while lowering the costs and risks of developing new drugs. CSCs exhibit remarkable metabolic plasticity across different tumor types and actively switch between glycolysis, lipid metabolism, amino acid metabolism and redox regulation to support self-renewal and resistance to therapy. This metabolic flexibility allows CSCs to survive changes in nutrients, oxygen and inflammatory signals within the TME but also reveals specific metabolic vulnerabilities.

This concept is increasingly supported by clinical trial data. An analysis of ClinicalTrials.gov showed that over 1,100 cancer-related trials included terms related to metabolism, with 71 specifically reaching phases 3–4. As of December 2025, data indicate that late-stage studies targeting glucose, lipid and amino acid metabolism, comprising 25, 10 and 17 phase 3–4 trials, respectively, are being recruited, highlighting the clinical maturity of metabolic intervention strategies. Notably, drugs originally developed for metabolic diseases, such as diabetes or hypertension, including metformin, pioglitazone and statins, have been widely repurposed for various cancer indications. Beyond pathway-based terms, many trials have mentioned that TME components are closely tied to CSC metabolic niches, such as TAMs, CAFs, neurons and TAAs. These patterns suggest that metabolic regulation is increasingly viewed in the context of tumor ecosystems rather than as an isolated cancer cell process.

Recent evidence suggests that the metabolic dependencies of cancer cells are strongly shaped by organ-specific microenvironments, which may partly explain the variable clinical efficacy observed in metabolic drug repurposing strategies. For example, metastatic breast cancer cells exhibit distinct nutrient requirements depending on the organ of colonization, indicating that metabolic vulnerabilities identified in one context may not be universally conserved across tumor sites^97^. At the same time, identifying robust metabolic targets remains challenging, as preclinical models often fail to recapitulate the metabolic landscape of patient tumors, particularly in metastatic settings, where organ-resident stromal and parenchymal cells influence nutrient availability and metabolic pathway utilization. Moreover, CSCs can readily activate salvage pathways or alternative metabolic routes in response to metabolic inhibition. Consistent with these complexities, recent translational studies highlight the difficulty of linking systemic metabolic interventions to clearly defined therapeutic mechanisms; for instance, fasting has been proposed to enhance endocrine therapy in estrogen-receptor-positive breast cancer through glucocorticoid-receptor-mediated transcriptional and epigenetic regulation, although the underlying mechanisms remain incompletely understood^98^. Collectively, these observations underscore the need for experimental strategies that more accurately capture the spatial and cellular complexity of tumor metabolism.

Future therapeutic strategies may therefore benefit from integrating organ-specific metabolic dependencies with tumor-intrinsic and microenvironmental contexts to develop more precise metabolic interventions. Rather than universally targeting metabolic pathways across tumor types, exploiting tissue-imposed metabolic constraints that shape CSC behavior may provide a more effective framework for precision metabolic therapy.

Importantly, repurposed metabolic drugs are most frequently evaluated in combination with standard chemotherapy, targeted therapy or immunotherapy rather than as monotherapies. This aligns with preclinical evidence showing that CSC metabolic dependencies are influenced by the context and shaped by therapeutic pressure and microenvironmental interactions. Overall, these data highlight that CSC metabolic vulnerabilities are not only biologically important but also clinically actionable, making drug repurposing a practical and scalable approach for translating these vulnerabilities into therapeutic benefits (Table 1).

**Table 1:** Repurposing of metabolic disease–related drugs for cancer therapy related to CSC metabolism (status as of December 2025).

Drug	Primary metabolic target	CSC-metabolism relevant mechanism	Cancer types	Affected TME component	Evidence level	NCT number
Metformin	Type 2 diabetes	OXPHOS inhibition, AMPK activation	Medulloblastoma, muscle-invasive bladder carcinoma, thyroid cancer	Organ-specific TME cell types	Phase 3, 4	NCT05230758, NCT06215976, NCT05468554
Statins	HMG-CoA reductase	Mevalonate/cholesterol biosynthesis, membrane biosynthesis	Melanoma, breast cancer,NSCLC	ECs, inflammatory niche	Phase 3, 4	NCT07303816, NCT06785974, NCT07254221, NCT04980716
Pioglitazone	Type 2 diabetes	Lipid metabolism, PPAR-γ agonist	Breast cancer	Systemic metabolic milieu, muscle	Phase 2, pilot	NCT05013255, NCT05753657
Ketorolac, celecoxib	COX inhibition	Inflammatory signaling	Breast cancer, metastatic liver cancer	Immune cell types	Phase 2	NCT06150898, NCT07174570
Tranexamic acid	Antifibrinolytic agent	Inflammation, ECM remodeling	Melanoma	Neutrophil, inflammatory niche	Phase 3	NCT05899465
Atovaquone	Mitochondrial respiration	Tumor hypoxia	Brain tumor	Hypoxia niche	Phase 1	NCT06624371
Artesunate	Antimalarial drug	Iron-dependent oxidative metabolism, ROS, redox buffering	Cervical cancer, colon cancer	Hypoxia niche	Phase 2	NCT07095478, NCT07095309
Haloperidol	Dopamine D2 receptor antagonist	Ferroptosis, autophagy inhibition	Recurrent GBM	Recurrent TME	Phase 2	NCT06218524

This table summarizes the key examples of drugs originally developed for metabolic or related chronic diseases that are now being repurposed for cancer treatment. The listed agents were categorized according to their primary metabolic targets (search terms: ‘Glucose metabolism’, ‘Lipid metabolism’, ‘Amino acid metabolism’ and ‘Ferroptosis’), impacted TME components (search terms: ‘TAM’, ‘CAF’, ‘Neuron’ and ‘Adipocyte’) and cancer indications. Drug-related terms were restricted to representative agents with established relevance to metabolic disease or metabolic targeting in cancer. Trials were filtered for ‘Not yet recruiting’ or ‘Recruiting’ status, and study phases were analyzed either across all phases or restricted to phase 3–4 trials as indicated. Duplicate or overlapping clinical trials were removed before the final analysis. The search cutoff date was December 2025. Clinical trial stages and corresponding ClinicalTrials.gov identifiers (NCT numbers) are displayed to show the current level of clinical evaluation.
